# Osteogenic lineage restriction by osteoprogenitors cultured on nanometric grooved surfaces: The role of focal adhesion maturation^☆^

**DOI:** 10.1016/j.actbio.2013.11.008

**Published:** 2014-02

**Authors:** John W. Cassidy, Jemma N. Roberts, Carol-Anne Smith, Mary Robertson, Kate White, Manus J. Biggs, Richard O.C. Oreffo, Matthew J. Dalby

**Affiliations:** a Centre for Cell Engineering, Institute of Biomedical and Life Sciences, Joseph Black Building, University of Glasgow, Glasgow G12 8QQ, UK; b Kelvin Nanotechnology, James Watt Nanofabrication Centre, University of Glasgow, Glasgow G12 8LT, UK; c Bone & Joint Research Group, Centre for Human Development, Stem Cells and Regeneration, Institute of Developmental Sciences, University of Southampton, Southampton SO16 6YD, UK; d Network of Excellence for Functional Biomaterials (NFB), National University of Ireland, Galway, Ireland

**Keywords:** Topography, Focal adhesion, Contact guidance, Bone formation, Tissue engineering

## Abstract

The differentiation of progenitor cells is dependent on more than biochemical signalling. Topographical cues in natural bone extracellular matrix guide cellular differentiation through the formation of focal adhesions, contact guidance, cytoskeletal rearrangement and ultimately gene expression. Osteoarthritis and a number of bone disorders present as growing challenges for our society. Hence, there is a need for next generation implantable devices to substitute for, or guide, bone repair in vivo. Cellular responses to nanometric topographical cues need to be better understood in vitro in order to ensure the effective and efficient integration and performance of these orthopedic devices. In this study, the FDA-approved plastic polycaprolactone was embossed with nanometric grooves and the response of primary and immortalized osteoprogenitor cells observed. Nanometric groove dimensions were 240 nm or 540 nm deep and 12.5 μm wide. Cells cultured on test surfaces followed contact guidance along the length of groove edges, elongated along their major axis and showed nuclear distortion; they formed more focal complexes and lower proportions of mature adhesions relative to planar controls. Down-regulation of the osteoblast marker genes RUNX2 and BMPR2 in primary and immortalized cells was observed on grooved substrates. Down-regulation appeared to directly correlate with focal adhesion maturation, indicating the involvement of ERK 1/2 negative feedback pathways following integrin-mediated FAK activation.

## 1 Introduction

By 2020 it is expected that the current number of patients suffering from bone diseases will double, with 9.3% of US adults predicted to suffer from osteoarthritis alone [1], [2]. Hence, there is a need to maximize the life expectancy of primary joint implants through understanding cell–material interactions.

It is becoming increasingly evident that the interface between host and implant is far more than a simple boundary of definition. Rather, host–implant interfaces provide a context for cellular adhesion and cell-specific orientation through first stage protein interactions and subsequent support of tissue neogenesis and cellular differentiation [3], [4]. The range of orthopedic biomaterials currently in use clinically typically lack in biofunctionality, resulting in micromotion of prosthesis after implantation and an increased risk of revision surgery. Aside from the clear morbidity and socio-economic implications, secondary surgery is accompanied by a twofold increased risk of further medical complications [5]. It is essential that future orthopedic devices are able to withstand micromotion by directing differentiation of locally derived mesenchymal (specifically skeletal) stem cells (MSCs) and osteoprogenitors (OPGs) into bone-matrix-secreting osteoblasts.

Contact guidance on grooved topographies is a well-documented cellular behaviour in vitro [6] and has biological relevance in vivo*.* The natural bone extracellular matrix provides an environment rich in both chemical and topographical cues capable of influencing and modulating cell behaviours. This macromolecular network of proteins, glycoproteins and polysaccharides is known to influence the differentiation of skeletal progenitor cells through focal adhesion formation, contact guidance, cytoskeletal rearrangement and ultimately gene expression [7], [8]. These are effects that we, and others, have replicated across a variety of cell types [7], [8], [9], [10], [11]. It is now recognized that capitalizing on topographical cues, alongside traditional chemical and biological signalling, is necessary to facilitate the successful integration and performance of a device in vivo.

A critical mechanism for cellular interaction with the extracellular matrix (ECM), and with biomaterials, is the process of cell adhesion. In addition to facilitating cellular tethering to substrata, focal adhesions form the basis of filopodia exploration of the ECM, subsequent lamellipodia ruffling and cellular spreading [12] in response to the extracellular landscape. As a cell spreads across a substrate, transient focal complexes (FX; adhesions of less than 1 μm) form along the cell’s leading edge, which may precede formation of larger focal adhesions (FA; 1–5 μm adhesions) [13].

Intrinsically linked to cellular spreading is the phenomenon of contact guidance, i.e. cellular tracing along the edge of a physical feature or obstacle. Contact guidance is associated with regular grooved substrata in Ref. [14] and drives processes such as the migration of immature neurons along scaffolds of glial fibres during development [15]. The use of grooved substrates provides a reproducible model to study the effects of focal adhesion formation in osteogenesis in vitro [17].

The fundamental component of the focal adhesion plaque and the receptor through which MSCs, osteoprogenitors and osteoblasts (amongst others) physically interact with the ECM is the integrin [16]. Integrins function in adhesion plaques by binding and mechanically coupling the cell’s cytoskeleton to the ECM and translating mechanical into biochemical signals within the cell; a process termed mechanotransduction [18]. In osteogenesis, focal adhesion-mediated mechanotransduction and integrin-dependent signalling pathways are dependent on non-receptor tyrosine kinases such as focal adhesion kinase (FAK) [19]. In addition to directly activating survival pathways (inhibiting anoikis and inactivating caspase 9 [20]), FAK promotes osteogenesis through extracellular signalling-related kinase 1 and 2 (ERK 1/2) pathways [21]. Through the integrin-dependent translocation of ERK, FAK mediates phosphorylation of the Runt-related transcription factor RUNX2 [22], the expression of which is highly restricted to skeletal tissue. RUNX2 acts as a master regulator in progenitor commitment to the osteoblast lineage and in transcription of osteoblast-specific genes essential for bone homeostasis (osteocalcin, osteopontin and matrix metalloproteinase 13 [7]). Through attachment to ECM elements, integrin clustering and the formation of actin stress fibres, focal adhesion formation and maturation are directly linked to FAK signalling, RUNX2 transcription and the induction of osteogenesis.

Here, we draw on the observation that as cellular spreading increases (either uniformly or as part of a polarized cellular morphology), tension placed on actin stress fibres anchored at focal adhesion plaques will result in a proportional and marked elongation of the adhesion itself through dynamic reinforcement [23]. We hypothesize that increased cellular motility caused by contact guidance on grooved substrates will inhibit osteogenesis through a reduction in adhesion maturation and reduced RUNX2 transcription. To investigate this, we have examined focal adhesion formation in response to 240 nm and 540 nm deep-grooved (12.5 μm wide) polycaprolactone (PCL) substrate, a biodegradable polyester approved by the US Food and Drugs Administration (FDA) for use in vivo. The osteoblast-like MG63 cell line and primary human osteoprogenitors cell populations (OPGs) were used in experiments. Comparisons were drawn between cells cultured on grooved and planar substrates and significance tested by statistical analysis of focal adhesion classification (FX, FA, SMA), proportions (%FX, %FA, %SMA) and total adhesion numbers. Focal adhesion orientation and nuclear distortion were quantified and polarized cellular morphologies were noted. Quantitative PCR was used to determine expression of osteoblast master transcription factor RUNX2 and related gene BMPR2 after 3 days of culture on planar control and groove substrates.

## 2 Materials and methods

### 2.1 Generation of grooved and planar growth surfaces

Grooved and planar slides were produced as previously described [24]. Planar slides were blanket-etched to ensure comparable chemistry to the grooved slides. Slide topographies were confirmed after original fabrication to be 12.5 μm × 240 nm and 12.5 μm × 540 nm. Quartz slides were used to create multiple polymer replicas by manual hot (80 °C) embossing polycaprolactone beads ((C_6_H_10_O_2_)*_n_*; PCL, washed in methanol for 1 week, Sigma Aldrich). Substrates were cooled on ice and trimmed to sizes suitable for cell culture in 24 well culture dishes: typically discs were 13 mm in diameter.

Wettability of PCL substrates was quantified by water contact angle analysis (WCA) with a KSV CAM 100 contact angle meter (KSV Instruments) using the sessile drop technique. Measurements were carried out in triplicate on three replicates of each sample. PCL substrates were treated for 30, 60, 90 or 120 s at MHz-range radio-frequency (RF) (10.15 W, 720 V DC, 15 mA DC) in a plasma cleaner (PDC-002, Harrick Plasma) to remove organic contaminants and improve cell surface attachment. Surfaces were sterilized in 70% ethanol for 1 h immediately following plasma treatment, followed by three sequential 5 s immersions in 70% ethanol, two sequential 5 s immersions in HEPES saline solution and a final 10 immersion in cell culture media prior to cell seeding.

### 2.2 Atomic force microscopy

Surfaces were analysed by atomic force microscopy (AFM) (scanning mode) after plasma treatment. The tip used was a non-conducting silicon nitride (MSCT, Veeco) with dimensions of 140 μm length by 18 μm width (resonance frequency 38 kHz, spring constant (*K*) 0.1 N m^−1^). Planar control substrates showed an average roughness of 18.5 nm over 100 μm^2^ (RMS 23.0 nm). Groove depths of 240 nm-grooved substrates were 210, 220 and 235 nm with an approximate width of 12.8 μm. 540 nm depths were 510, 530 and 535 nm and width ∼13.1 μm.

### 2.3 Cell isolation and culture

OPGs were isolated from haematologically normal patients undergoing routine surgery as previously described [25]. Only tissues that would have been discarded were used and only with the approval of the Southampton and South West Hants Local Research Ethics Committee. OPGs were cultured in growth media containing 88% Dulbecco’s modified Eagle’s medium (DMEM; Sigma) supplemented with 8.8% fetal bovine serum (FBS; Lonza), 1.5% penicillin streptomycin, 0.85% 100 × non-essential amino acids (Invitrogen) and 0.85% 100 mM sodium pyruvate (Life Technologies). The medium was changed every 3 days and cells were passaged as required by trypsinization. MG63 human osteoblast-like cells were cultured in media containing 71% DMEM, 17.5% Medium 199 (Sigma), 10% FBS, 1% penicillin streptomycin and 0.5% 100 mM sodium pyruvate. Cells were incubated at 37 °C with a 5% CO_2_ atmosphere and serum starved for 24 h prior to seeding to encourage populations with synchronized cell cycles. Cells were seeded at 1 × 10^4^ cells ml^−1^ in assay media containing 1% FBS.

### 2.4 Coomassie blue histology and cell counts

After 72 h, cells were washed in PBS and fixed with 4% formaldehyde/PBS. Cells were stained for 2 min in 0.5% Coomassie blue in a methanol/acetic acid aqueous solution and washed with ultrapure water to remove excess dye. Digital images were captured using a Zeiss Axiovert 25 under light microscopy. Cells were counted in three random fields of view for each of three replicates.

### 2.5 Immunocytochemistry

After 3 days of culture, cells on test surfaces were fixed in 3.75% paraformaldehyde and permeabilizing buffer (10.3 g of sucrose, 0.292 g of NaCl, 0.06 g of MgCl_2_, 0.476 g of HEPES and 0.5 ml of Triton X-100 in 100 ml of H_2_O at pH 7.2) was added for 5 min at 4 °C. Samples were then incubated for 5 min at 37 °C in 1% BSA/PBS to block non-specific binding. Anti-vinculin primary antibody (1:150 in 1% BSA/PBS, hVin1 monoclonal antihuman raised in mouse (IgG1), Sigma) or anti-paxillin (1:150 in 1% BSA/PBS, pY31 monoclonal antihuman raised in mouse (IgG1), BD Biosciences) and rhodamine-conjugated phalloidin (1:50 in 1% BSA/PBS, Molecular Probes) were added for 1 h at 37 °C. Substrates were washed thrice in 0.5% Tween 20/PBS (5 min each). A secondary, biotin-conjugated antibody (1:50 in 1% BSA/PBS monoclonal horse antimouse (IgG), Vector Laboratories) was added for 1 h at 37 °C followed by substrate washing as described above. FITC-conjugated streptavidin was added (1:50 in 1% BSA/PBS, Vector Laboratories) for 30 min at 4 °C before samples were given a final wash. Surfaces were mounted using mounting medium for fluorescence, with DAPI counterstain (Vector Laboratories), and viewed by fluorescent microscopy (Zeiss Axiophot). Digital images were captured in three fluorescent channels (×100 magnification) and saved for further processing.

### 2.6 Focal adhesion analysis

**Fig. 1:**
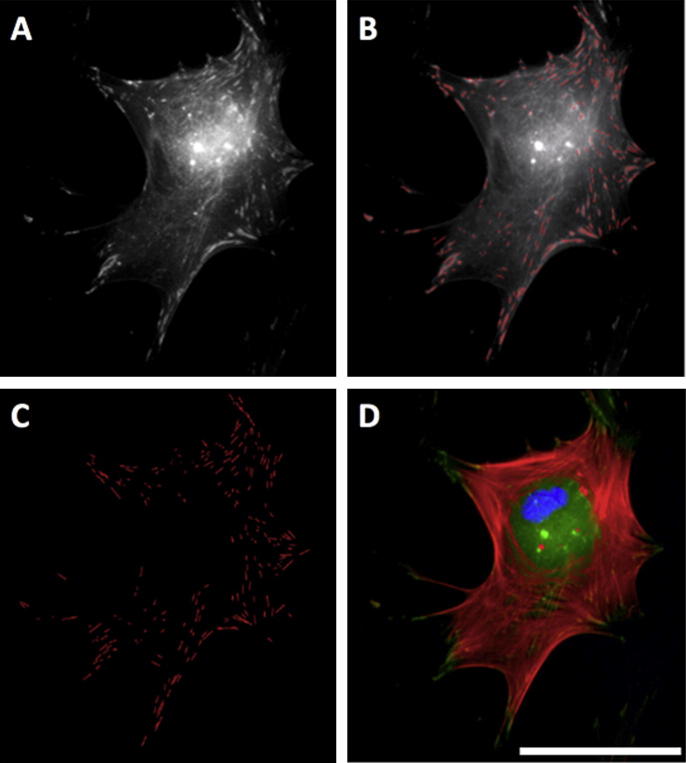
Focal adhesion analysis (OPG cells). Greyscale images of immunolabelled adhesions were exported to Adobe Photoshop (panel A) and each identified adhesion traced with a 1-pixel straight line, creating an adhesion mask superimposed over the background image (panel B). Care was taken to avoid labeling bleed from the actin channel. The background image was removed (panel C) and the adhesion mask exported to ImageJ for analysis. Panel D shows a complete RGB image: red is actin, green vinculin and blue DAPI (nuclear). Bar is 25 μm.

**Supplementary Figure 1:**
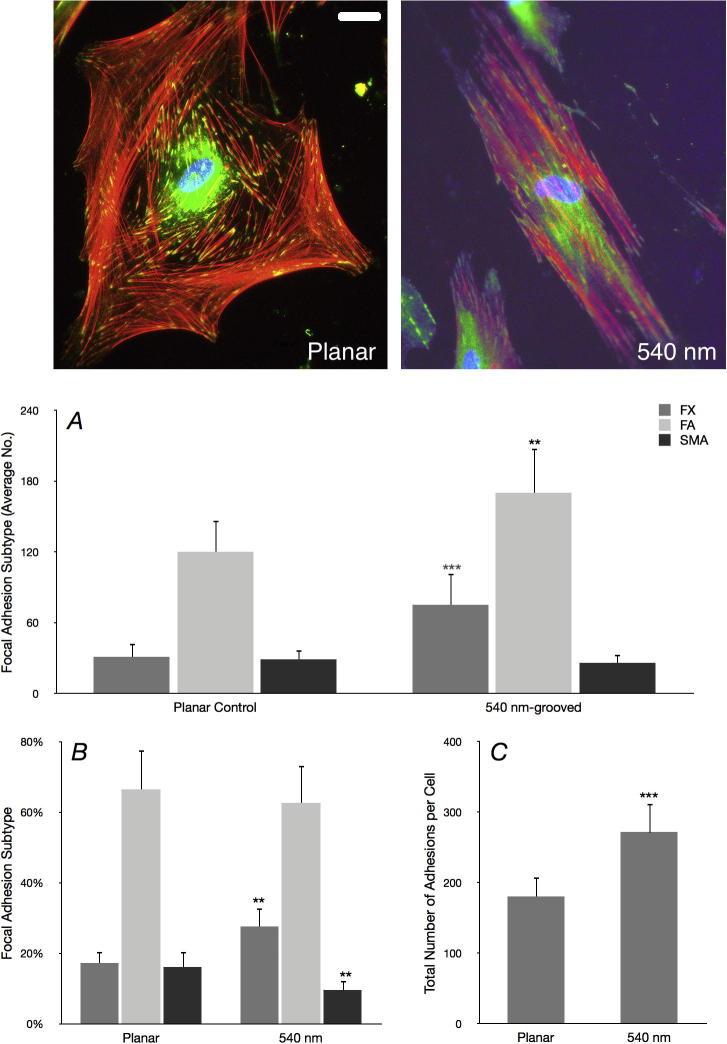
Validation of focal adhesion analysis (OPG cells). Greyscale images of immunolabelled adhesions labeled for *paxillin* were exported to Adobe Photoshop, and adhesions characterized as described in Fig. 1. Top panel shows RGB composite images of cells cultured on either planar or 540 nm-grooved PCL substrates. Red is actin, green paxillin and blue is nuclear; bar is 10 μm. Bottom panel shows focal adhesion subtypes and percentage subtype normalized to cell numbers (40 distinct cells). Relative proportions were similar to vinculin-based analysis, validating the use of vinculin as an adhesion marker. Statistical significance (*t-*test) relative to planar is noted (^*^p < 0.05, ^**^p < 0.01 and ^***^p < 0.001).

Greyscale images of adhesions were exported to Adobe Photoshop (Fig. 1, panel A), and each identified adhesion traced creating a superimposed adhesion mask (Fig. 1, panel B). Care was taken to avoid labelling fluorescent bleed from the actin channel. The background image was removed (Fig. 1, panel C), and the adhesion mask exported to ImageJ (http://rsbweb.nih.gov/ij/) to calculate adhesion numbers and lengths. Adhesions were characterized according to size, as described by Bershadsky et al. [12] and Biggs et al. [27]. Briefly, adhesions measuring less than 1 μm were classed as focal complexes (FXs), 1–5 μm as focal adhesions (FAs) and greater than 5 μm as supermature adhesions (SMAs). Those greater than 8 μm were classed as large SMAs and those greater than 10 μm as very large SMAs. For each condition, three biological replicates (of 40 distinct cells) were analyzed. Staining for paxillin, Supplementary Fig. S.1, validated the use of vinculin as a focal adhesion marker, showing similar changes in focal adhesion length independent of marker protein*.* Adhesions were normalized to cell number rather than circumference to avoid changes due to cell cycle.

### 2.7 Quantification of alignment

Images captured in the DAPI channel were exported to ImageJ for analysis. 1-pixel wide lines were traced over long and short axes of nuclei from 40 distinct cells per condition. The length of each axis was calculated in ImageJ, after which a ratio representing longest over shortest was calculated. Values of 1 represent a perfectly spherical nuclei and >1 nuclei elongated along the major axis. Standard deviations of adhesion angles were also calculated (excluding FX angles) and values taken as arbitrary measures of adhesion angle variance. A higher standard deviation (SD) corresponds to FA and SMA adhesions formed in a uniformly random pattern, whereas a low SD corresponds to more aligned adhesions, typical seen around the cell’s polar extremes. The Ward and Hammer model of adhesion growth shows that as a focal adhesion is put under tension from actin stress fibres, the plaque will elongate in an anisotropic manner [23], [28], [29]. Thus, adhesion orientation is intrinsically representative of cytoskeletal tension and a good measure of cellular polarization and morphology. For each condition, three biological replicates (of 40 distinct cells) were analysed.

### 2.8 Quantitative PCR

After 72 h culture, cells were lysed and total RNA extracted using an RNeasy kit (Qiagen) according to the manufacturer’s instructions, noting that lysates from four surfaces were pooled to form one replicate (of which there were three per condition). Reverse transcription was carried out on extracted RNA using an Omniscript Reverse Transcription kit (Qiagen) according to the manufacturer’s instructions, with Random Primers (Invitrogen) and RNAsin (Promega). Quantitative PCR was carried out using a qPCR detection system (model 7500, Applied Biosciences) by the SYBR green method. Runt related protein 2 (RUNX2) and bone morphogenic protein receptor type 2 (BMPR2) were probed for with commercially available primers (Applied Biosystems). Primers sequences were BMPR2 forward 5′-CAGACCAGCAGCACTCCATA-3′ and reverse 5′-CAGCGTCAACACCATCATTC-3′; RUNX2 forward 5′-GTGCAGAGTCCAGCAAAGGT-3′ and reverse 5′-TCAGCCAACTCGTCACAGTC-3′. GAPDH expression was used as a reference gene to normalize all data. RQ (relative gene expression) values were automatically calculated by the delta delta CT method. Statistical analysis first determined that GAPDH did not vary under test conditions (one-way ANOVA; OPG *p* = 0.61; MG63 *p* = 0.67). Cycle threshold values were then converted from logarithmic to linear scale (2^-^*^CT^*) for further analysis.

### 2.9 Oligo GEarray

Following 72 h culture, cells were lysed and total RNA was isolated from cells on grooved and control substrates by TRIZol reagent (Invitrogen, Carlsbad, CA), and its concentration and integrity were determined spectrophotometrically. The Oligo GEarray microarray series (SA Biosciences) were used to quantify the expression of 113 genes associated with endothelial cell biology. This technique is highly specific (90% homology) and has an inter-assay coefficient of variation <10%. Briefly 3 μg total RNA was reverse transcribed into Biotin-16-dUTP-labelled cDNA probes with the TrueLabeling-AMP method (SA Biosciences) according to the manufacturer’s instructions. The Human Osteogenesis (OHS-026) SuperArray membranes were pre-hybridized at 60 °C for at least 2 h. Hybridization of the Biotin-labeled cDNA probes to the membranes was carried out at 60 °C overnight with slow agitation in a hybridization oven. The hybridized membranes were washed in saline sodium citrate buffer once in solution I (one in 2× SSC, 1% SCS) and once in solution II (0.1 × SSC, 0.5% SDS). For detection, membranes were incubated with alkaline phosphatase-conjugated streptavidin, washed and incubated with the chemiluminescent substrate CDP-Star. Membranes were exposed to X-ray film (Kodak, XAR film) and analysed with ImageJ software. The relative expression level of each gene was determined by comparing the signal intensity of each gene in the array after correction for background and normalization.

### 2.10 Statistical analysis

All statistical analyses were carried out in Microsoft Excel 2010. Student’s *t*-test (for two samples, assuming unequal variances, two-tailed) or one-way ANOVA was used to calculate if observed differences between test and control surfaces were statistically significant (differences: ^*^p < 0.05, ^**^p < 0.01 and ^***^p < 0.001 denoted). All values quoted are mean ± standard deviation.

## 3 Results

### 3.1 PCL cell culture optimization

**Fig. 3:**
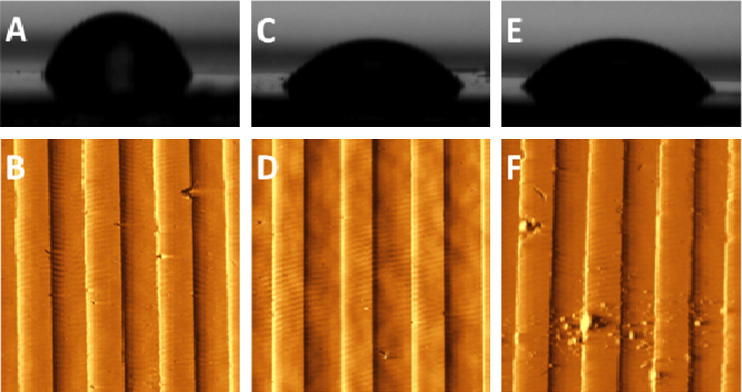
Surface optimization. Water contact angle analysis (panel A) and AFM surface imaging (panel B) of untreated 540 nm grooved controls and the same surface after 30 s (panels C and D) and 2 min plasma treatment (panels E and F). Panel F shows groove integrity compromised, thought to be due to prolonged plasma treatment.

**Fig. 4:**
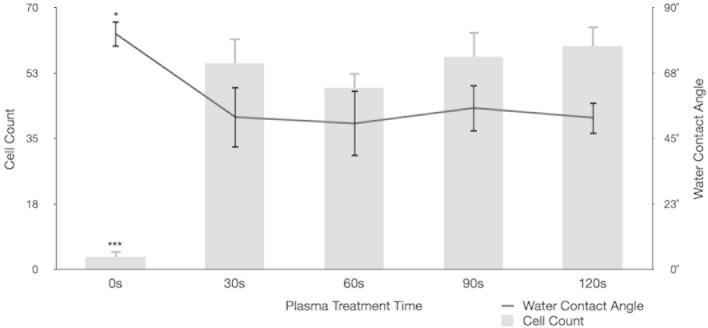
Cellular growth assays. Water contact angle analysis of surfaces after various plasma treatment times (trendline; right axis) with corresponding MG63 cell counts at 24 h (bar chart; left axis). There is a marked and statistically significant (^***^p *<* 0.001) fall in surface energy after 30 s treatment, as seen by WCA, and a corresponding increase in 24 h MG63 cell count over 10 random fields of view (^***^p *<* 0.001).

MG63’s initially displayed poor growth and high levels of apoptosis when cultured on both test and control substrates. Adherent cells are known to require integrin signalling by attachment to the ECM in order to avoid anoikis, to proliferate and to undergo differentiation [30]. Osteoblast-like cells, in particular, show high levels of apoptosis and low levels of proliferation following culture on hydrophobic surfaces – a feature thought to be due to defective Ras activation by fibroblast growth factor 1 (FGF1) [30]. As the chemical composition of PCL is rich in uncharged elements [31] it was reasoned that substrates were too hydrophobic to support cell growth. Water contact angle analysis of PCL substrates confirmed they were highly hydrophobic (Fig. 3, Fig. 4). Plasma treating surfaces for 30 s at a medium radio frequency (RF; 10.15 W) showed a marked decrease in PCL hydrophobicity (Fig. 3, Fig. 4). Following plasma treatment groove dimensions were found unchanged after the maximum treatment duration (2 min) (Fig. 3, panels C and D). Water contact angle decreased significantly (^*^p < 0.05) after a brief 30 s treatment, from 80.9 ± 4.6° to 52.3 ± 10.6°, and was unchanged thereafter – indicating that a treatment time of 30 s may be sufficient to permit cellular attachment and growth (Fig. 4). Cell counts of three random fields of view per replicate showed significantly increased cell density on all treated surfaces relative to untreated controls (^***^p < 0.001) after 24 h. Treating surfaces for longer than 30 s did not increase cell density further.

### 3.2 Focal adhesion analysis

**Fig. 5:**
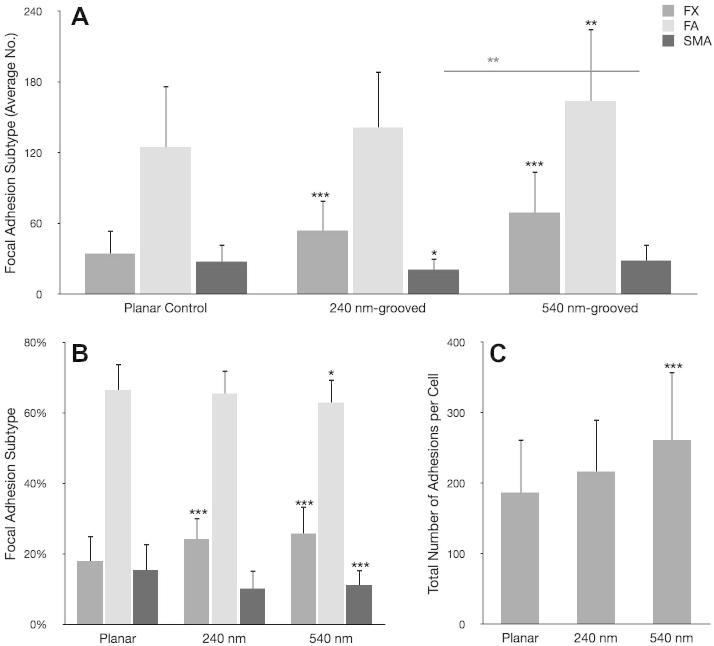
Focal adhesion analysis (OPGs). Adhesion analysis of OPG cells cultured on test and control substrates, statistical significance (*t*-test) relative to planar controls is noted (^*^p < 0.05, ^**^p < 0.01 and ^***^p < 0.001). Panel A shows absolute adhesion subtype counts, panel B shows the percentage of each subtype and panel C shows total adhesion counts. In addition to differences relative to controls, the absolute numbers of SMAs between cells cultured on 540 nm and 240 nm grooves was found to be statistically significant (^**^p < 0.01). Adhesion classification rationale can be found in the methods (FX<1 μm<FA<5 μm<SMA).

**Fig. 6:**
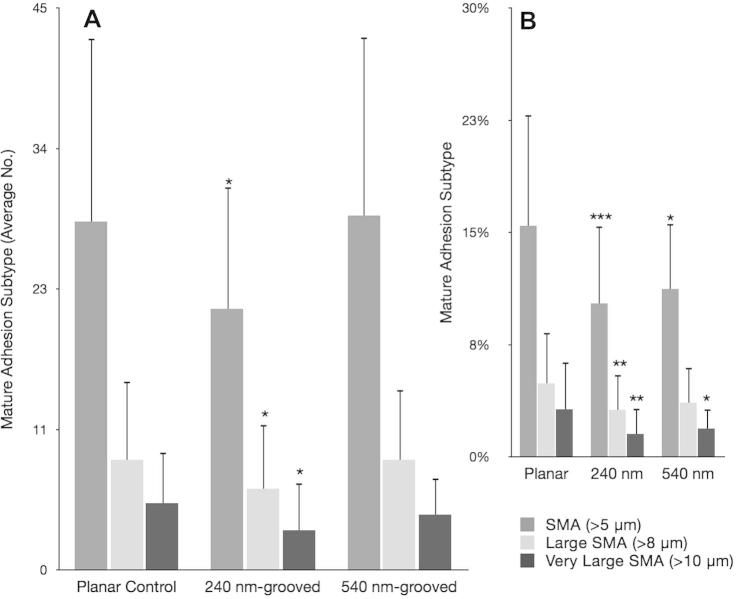
Mature adhesion analysis (OPGs). Graphs show mature adhesion breakdown of primary osteoprogenitors on test and control surfaces. In addition to super mature adhesions (SMAs > 5 μm), these data distinguish large SMAs (SMAs > 8 μm) and very large SMAs (>10 μm). Panel A shows absolute counts whereas panel B shows the proportion of each subtype. Statistical significance (*t*-test) relative to planar controls is noted (^*^p < 0.05, ^**^p < 0.01 and ^***^p < 0.001).

**Fig. 7:**
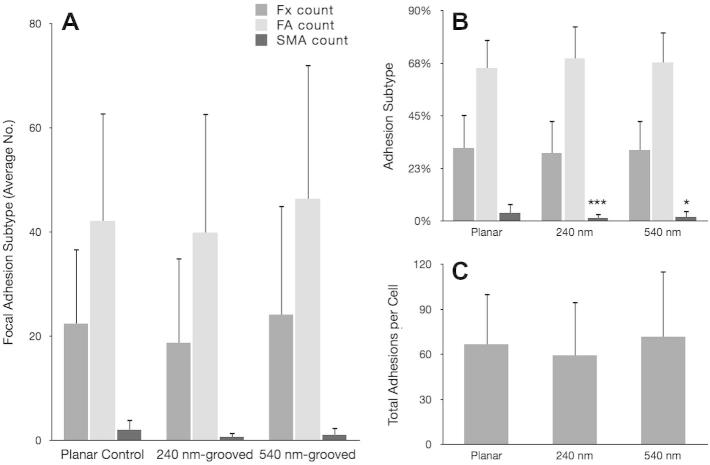
MG63 focal adhesion analysis*.* Focal adhesion analysis of MG63 immortalized osteoblast-like cells cultured on test and control substrates. Panel A shows absolute adhesion subtype counts, panel B shows the percentage of each subtype and panel C shows total adhesion counts. Adhesion classification rationale can be found in the methods (FX < 1 μm < FA < 5 μm < SMA). Statistical significance (*t*-test) relative to planar controls is noted (^*^p < 0.05, ^**^p < 0.01 and ^***^p < 0.001).

Vinculin is present in all focal adhesion subtypes; it provides a useful detection method for focal adhesions in general [26], [27]. This study used specific antibody detection to probe for vinculin and subsequently classify focal adhesion subtypes based on length and orientation. To validate the use of these observations, we also stained for the focal adhesion protein paxillin and found truncation of focal adhesion length independent of the marker used, Supplementary Fig. S.1. Analysis showed that OPGs cultured on grooved substrates formed significantly more FXs (^***^p < 0.001) than OPGs cultured on planar controls (240 nm formed 54 ± 12 per cell and 540 nm formed 69 ± 35 per cell, planar formed 34 ± 20 per cell) (Fig. 5). They also formed greater numbers of FAs (planar 124 ± 52 per cell, 240 nm 141 ± 48 per cell and 540 nm 163 ± 61 per cell). However, the total number of adhesions formed by cells cultured on grooved substrates increased by proportionally (planar 187 ± 75 per cell, 240 nm 216 ± 74 per cell and 540 nm 261 ± 96 per cell), resulting in relatively similar FA proportions on each surface (planar 66 ± 7% FA, 240 nm 65 ± 7% FA and 540 nm 63 ± 7% FA). SMA formation was significantly down-regulated on 240 nm grooves (^*^p < 0.05), but not on 540 nm grooves (*p* = 0.878) where SMA numbers were similar to cells cultured on planar control substrates (planar 28 ± 15 per cell, 240 nm 21 ± 10 per cell and 540 nm 28 ± 14 per cell) (Fig. 5, Fig. 6). In contrast to FA proportions, the increase in total adhesion number seen on test surfaces translated into a significantly reduced proportion of SMAs relative to controls (planar 15 ± 7%, 240 nm 10 ± 5% and 540 nm 11 ± 4%; 240 nm ^***^p < 0.001; 540 nm ^**^p < 0.01). Following this pattern, OPGs cultured on planar controls had significantly greater proportions of large SMAs and very large SMAs than those cultured on 240 nm (^**^p < 0.01 in each case) and 540 nm (^*^p < 0.05 in each case) grooved substrates, though 540 nm had similar absolute values to controls. To confirm these data, MG63s were also analysed (Fig. 7), and showed a similar, but less pronounced trend to primary cells.

### 3.3 Cellular orientation and motility

**Fig. 2:**
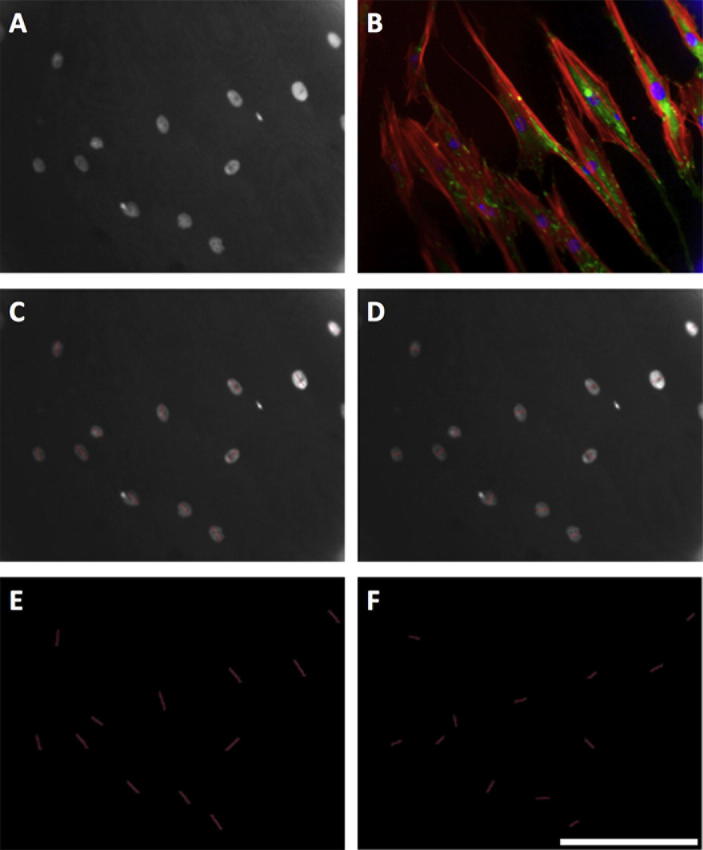
Nuclear distortion analysis (OPG cells). Greyscale images captured in the DAPI channel (panel A) were exported and nuclei traced along their long and short axes (panels C and D). Background images where removed (panels E and F) and axis lengths calculated in ImageJ. The corresponding ratio gives a measure of nuclear elongation along the major axis. Panel B shows a complete RGB image: red is actin, green vinculin and blue DAPI (nuclear). Bar is 50 μm.

**Fig. 8:**
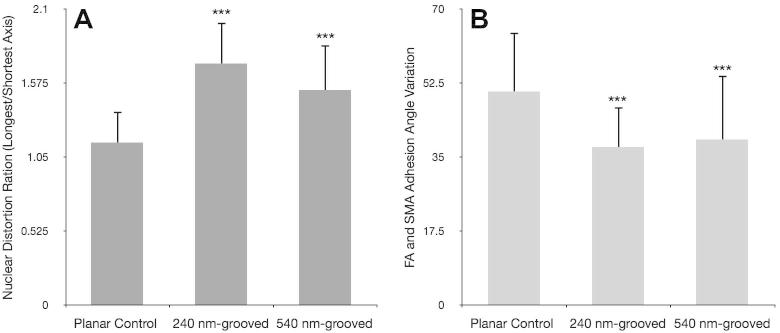
Relative focal adhesion alignment and nuclear distortion (OPGs). Focal adhesion alignment quantified via FA and SMA angle standard deviation (panel B). Nuclear distortion is presented as a ratio of longest over shortest axis, higher values indicate elongated nuclei (panel A). Statistical significance (*t*-test) relative to planar is noted (^*^p < 0.05, ^**^p < 0.01 and ^***^p < 0.001). Cells cultured on grooved surfaces showed significantly more elongated nuclei and less variant adhesion angles relative to those on planar controls. 540 nm-grooved surfaces tended to correspond to more angle variation and less distorted nuclei than 240 nm-grooved surfaces, though this trend was not significant.

Nuclear alignment was significantly increased when cells were cultured on grooved substrates (^***^p < 0.001), (see Fig. 2). Primary cells cultured on 240 nm and 540 nm grooves had an average maximum/minimum axis ratio of 1.7 ± 0.3 and 1.52 ± 0.3, respectively, compared to 1.15 ± 0.2 on planar controls (^***^p < 0.001 in each case). On average, nuclear length was more than 1.5 times greater than nuclear width when cells were cultured on either of the grooved substrates. MG63 cells showed similar nuclear elongation, and in each case planar surfaces encouraged growth of cells with a ratio closer to 1.0 (length = width) (Fig. 8)*.* Similarly, adhesion (FA and SMA) orientation was less variable in cells cultured on grooved substrates (^***^p < 0.001; ANOVA). Planar angle variation was 50.5 ± 14.1, 240 nm was 37.3 ± 9.5 and 540 nm was 39.2 ± 15.3 (Fig. 8). Angle variance between cells on different groove depths were not significant (*p* = 0.522; ANOVA), though there was a trend indicating less well aligned adhesions on 540 nm grooves. All test conditions were highly significantly different from controls (^***^p < 0.001). Additionally, it was noted that 65.5% of cells cultured on 240 nm deep grooves, 65.8% on 540 nm, but only 41.5% of cells on planar controls, contained 20 or more focal complexes or small focal adhesions (<2.5 μm) at the perinuclear area.

### 3.4 Quantitative PCR

**Fig. 9:**
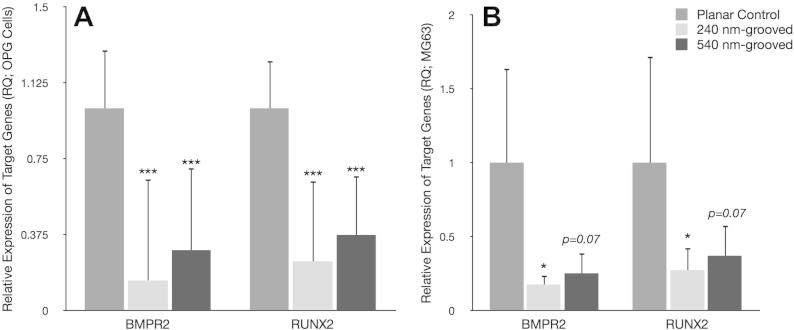
Relative expression of target genes BMPR2 and RUNX2 (OPGs). Values presented are RQ (expression relative to planar control) and have been normalized to housekeeping gene GAPDH by the delta delta CT method. Each experiment consisted of three replicates per condition and each replicate was the result of four pooled samples. Both BMPR2 and RUNX2 expression was significantly (^***^p < 0.001 and ^**^p < 0.01 respectively) down-regulated in both test conditions relative to planar controls. Expression of target genes was down-regulated to a greater extent on 240 nm compared to 540 nm-grooved substrates in each experiment, though this trend was not significant. MG63 down-regulation was significant (^*^p < 0.05) on 240 nm substrates but not on 540 nm (RUNX2 *p* = 0.07; BMPR2 *p* = 0.07) (panel B).

**Supplementary Figure 2:**
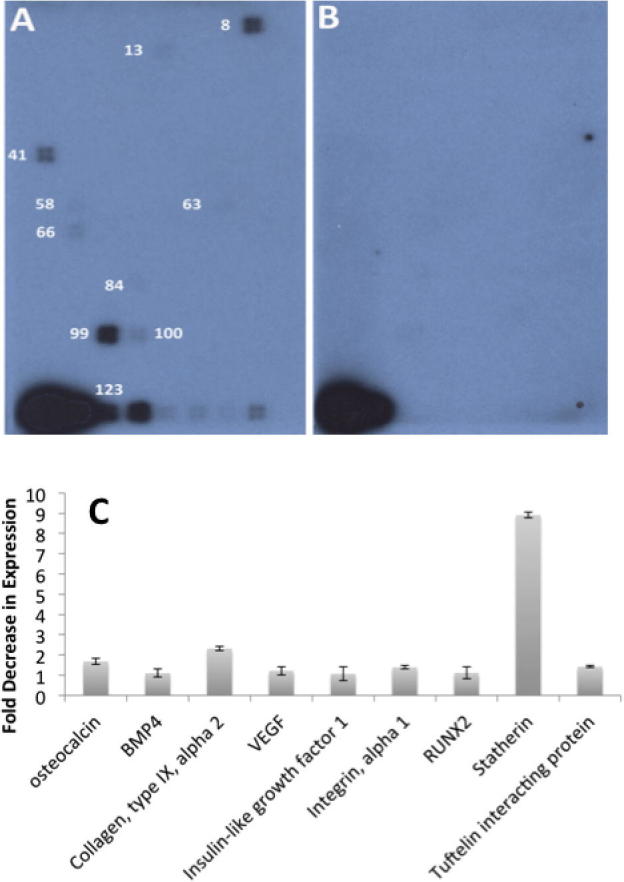
Protein data from Oligo GEarrays (Stro-1 MSC cells). Fig. shows bone-related gene expression of MSC cells cultured on 10 μm wide and 330 nm deep grooved surfaces, embossed into polymethylmethacrylate (PMMA) with the arrays performed as per the manufacturer’s instructions. Panel A shows control and panel B shows 330 nm grooves. Panel C shows fold decrease in gene expression. 8 is osteocalcin (non-collagenous protein found in bone); 13 is BMP4 (bone morphogenic protein 4, involved in bone formation); 41 is collagen, type IX, alpha 2; 58 is VEGF (vascular endothelial growth factor, angiogenesis during bone formation); 63 is insulin-like growth factor 1; 66 is Integrin, alpha 1; 84 is RUNX2; 99 is Stratherin; 100 is Tuftelin interacting protein 11 (involved in mineralization process). 123 is GAPDH control.

QPCR showed significant down-regulation of both RUNX2 and BMPR2 expression in OPGs when cultured on grooved substrates (^***^p < 0.001 and ^**^p < 0.01 respectively) relative to planar controls. Moreover, expression of RUNX2 or BMPR2 was not significantly different between the two grooved depths assayed. Average down-regulation of RUNX2 in MG63s was approximately fourfold (^*^p < 0.05) on 240 nm grooves and threefold (*p* = 0.06) on 540 nm grooves; BMPR2 was fivefold (^*^p < 0.05) and fourfold, respectively (*p* = 0.07) (Fig. 9). In each qPCR assay it was observed that BMPR2 was down-regulated to a greater degree than RUNX2. Though this trend was not significant, it could offer insight into the proposed role of each in osteogenesis [7]. Oligo GEarrays, Supplementary Fig. S.2, confirmed down-regulation of RUNX2 when cells were cultured on grooves, along with various other bone markers. Although protein level down-regulation of bone markers, such as osteocalcin, would be useful, this work has not yet been undertaken.

## 4 Discussion

The current study compared relative and absolute focal adhesion number, size and orientation for cells cultured on grooved and planar control substrates. Nuclear polarization and relative expression of BMPR2 and RUNX2 were quantified for test and control surfaces with both primary OPG and MG63 cells. The PCL substrates used in this study were unable to support cell growth untreated, due to their composition of uncharged elements and resultant hydrophobicity [30], [31]. This problem was negated by oxygen-plasma-treating surfaces for 30 s. Treated surfaces showed a marked decrease in hydrophobicity yet surface dimensions were unchanged and groove integrity was unaffected. Treated surfaces supported significantly increased growth of MG63 cells (Fig. 3).

Cells cultured on planar controls showed a near uniformly spread morphology with significantly fewer focal complexes (associated with cellular spread) and more mature adhesions than on grooved substrates. Adhesion angles showed significantly increased variance on planar substrates compared to grooves, consistent with the observed non-polarized cellular morphology. These data are consistent with non-motile cells, unaffected by contact guidance, which are spreading uniformly and increasing cytoskeletal tension on their focal attachments, leading to the formation of SMAs. Adhesion elongation is thought to follow the model put forward by Ward and Hammer [28] and Reinhart-King et al. [29]. In this model, tension causes the elongation of the adhesion plaque in an anisotropic manner, i.e. in the direction of the applied force [23]. It follows that an elongated focal adhesion would trigger the recruitment of more integrin receptors to anchor it to the ECM. Integrin receptor signalling is mediated by non-receptor tyrosine kinases, the most significant of which is FAK [21]. FAK promotes cell survival and osteogenesis through the ERK 1/2 pathways [19] and transcription of osteoblast-specific genes essential for bone homeostasis [7], as has been described.

Cells cultured on planar controls did not undergo contact guidance, resulting in greater cellular spread and more mature adhesions. It is thought that recruitment of integrin receptors occurred and downstream FAK/ERK 1/2 signaling led to the observed up-regulation of RUNX2 and BMPR2 relative to cells cultured on grooves. To support this hypothesis, further studies should inhibit FAK/ERK signalling and confirm relative down-regulation of RUNX2. Supplementary Fig. S.2 shows down-regulation of integrin α1 when MSCs are cultured on 330 nm grooves, which would seem to support this hypothesis, if not the precise signalling pathways.

Cells cultured on 240 nm and 540 nm deep (12.5 μm wide) grooved substrates were observed to follow contact guidance along the groove length. This resulted in a more polarized cellular morphology, reflected in both initial observations and by decreased adhesion angle variance relative to planar control. Additionally, cells cultured under test conditions developed more focal complexes (FX); adhesions associated with cellular motility and cells cultured on 240 nm grooves developed significantly less SMAs. It seems likely that reduced adhesion maturation and elongation trough molecular reinforcement results in reduced FAK activation/phosphorylation, perturbing the onset of osteogenesis through activation of the RUNX2 master transcription factor [19], [37]. This hypothesis fits with what is currently understood of focal adhesions and their downstream signalling; it also fits the data presented.

Cells cultured on 540 nm deep grooved surfaces showed similar SMA numbers to planar controls; significantly more than seen on 240 nm deep grooved substrates (^**^p *<* 0.01). Deeper grooves appeared to increase FA maturation; however, nuclei in cells cultured on these deeper grooves were significantly less polarized (^***^p < 0.001) than cells cultured on 240 nm grooves and adhesion angle variance was slightly higher (though not significantly). It would appear that 540 nm deep grooved substrates cause a slight decrease in cellular polarization, allowing for cell spread along their minor axis and hence permitting promotion of adhesion maturation. It is worth noting, however, that no differences in morphology were obvious between cells cultured on 240 nm and 540 nm grooved surfaces. Overall, cells cultured on grooved substrates formed more adhesions, in agreement with previous work [32], [33].

Our favoured hypothesis to account for the results presented is that whilst migrating cells would have baseline ERK 1/2 cytoplasmic activity to support proliferation, as focal adhesions mature FAK activation of ERK would cause both integrin-dependent translocation of ERK to the nucleus and cytosolic negative feedback. ERK 1/2 is known to have differing cytoplasmic and nuclear phosphorylation targets [34], [35]. Acting as a downstream element in Ras and receptor tyrosine kinase (RTK) signalling, ERK is associated with cellular growth and proliferation through regulation of growth factor responsive elements in the cytosol [35]. As ERK 1/2 is localized to the cytoplasm in this scenario, it follows there would be an associated down-regulation of nuclear c-Myc and increased expression of N-Myc [36]. Myc modulation in this manner causes a decrease in osteocalcin expression and an increase in focal adhesion turnover [37], favouring the formation of small transient FXs and inhibiting osteogenesis in migratory cells. This is consistent with observations of cells following contact guidance on 540 nm and 240 nm deep grooved substrates, which form a significantly lower proportion of SMAs than planar controls. However, kinase inhibition studies would have to be carried out before this hypothesis could be expanded upon, or confirmed.

As a progenitor cell ceases to migrate, focal adhesions mature and subsequent integrin clustering on the adhesion plaque would lead to FAK-mediated activation of ERK, through relatively well-defined pathways [19], [37]. Further to previous work carried out by this group [38], it is postulated that increased ERK 1/2 activation by these mechanisms is sufficient to activate the ERK negative feedback pathway, perhaps by phosphorylation of SOS (Son of Sevenless) [39]. Active ERK still in the cytosol will then translocate to the cell’s nucleus by adhesion-dependent Rac activation [40]. Here ERK can phosphorylate RUNX2, committing the cell to osteoblastic lineage while a decrease in cytosolic ERK will reduce sensitivity to growth factors [37].

However, from the ERK1/2 negative feedback hypothesis outlined above, it would follow that as cells cultured on 540 nm deep grooved substrates contain similar numbers of SMAs and more FAs than planar controls, FAK signalling should be up-regulated as more integrin receptors are recruited to adhesion plaques. ERK 1/2 signalling and nuclear translocation would thus lead to RUNX2 phosphorylation and activation in cells cultured on 540 nm deep grooved substrates. Although we have shown a marginal increase in RUNX2 and BMPR2 expression in cells cultured on 540 nm deep grooves over 240 nm deep, this trend is not significant.

## 5 Conclusions

The current results show a significant increase in both total numbers of focal adhesions and a decrease in average focal adhesion length on cells cultured on grooved substrates relative to planar controls. Relative to total number of adhesions, cells cultured on grooved substrates had fewer SMAs and more FXs than planar controls. Highly polarized cell morphologies were also observed on cells cultured on grooved substrates. Significant differences were found between expression of osteogenic markers RUNX2 and BMPR2, which were both down-regulated by grooved surfaces relative to controls. The study further delineates the role of adhesions in osteogenesis and contact guidance.

## Appendix B Figures with essential colour discrimination

Certain figures in this article, particularly Figs. 1–3, are difficult to interpret in black and white. The full colour images can be found in the on-line version, at http://dx.doi.org/10.1016/j.actbio.2013.11.008.
